# Copeptin Levels Are Independent from Mild Therapeutic Hypothermia but Do Not Predict Infarct Size in Patients Presenting with ST-Segment Elevation Myocardial Infarction

**DOI:** 10.3390/jcdd8100131

**Published:** 2021-10-14

**Authors:** Matthias Mueller, Dietrich Beitzke, Thomas Scherz, Christian Loewe, Andreas Mangold, Rodrig Marculescu, Michael Poppe, Fritz Sterz, Harald Herkner, Irene Lang, Christoph Testori, Christoph Weiser

**Affiliations:** 1 Department of Emergency Medicine, Medical University of Vienna, 1090 Vienna, Austria; matthias.mueller@meduniwien.ac.at (M.M.); michael.poppe@meduniwien.ac.at (M.P.); fritz.sterz@meduniwien.ac.at (F.S.); harald.herkner@meduniwien.ac.at (H.H.); christoph.weiser@meduniwien.ac.at (C.W.); 2 Division of Cardiovascular and Interventional Radiology, Department of Biomedical Imaging and Image-Guided Therapy, Medical University of Vienna, 1090 Vienna, Austria; dietrich.beitzke@meduniwien.ac.at (D.B.); christian.loewe@meduniwien.ac.at (C.L.); 3 Division of Cardiology, Department of Internal Medicine II, Medical University of Vienna, 1090 Vienna, Austria; thomas.scherz@meduniwien.ac.at (T.S.); andreas.mangold@meduniwien.ac.at (A.M.); irene.lang@meduniwien.ac.at (I.L.); 4 Department of Laboratory Medicine, Medical University of Vienna, 1090 Vienna, Austria; rodrig.marculescu@meduniwien.ac.at

**Keywords:** copeptin, arginine vasopressin, acute myocardial infarction, targeted temperature management, mild therapeutic hypothermia

## Abstract

Background: Mild therapeutic hypothermia (MTH) is a treatment adjunct in ST-segment elevation myocardial infarction (STEMI) that deserves investigation. Copeptin―a surrogate marker for vasopressin―is an early biomarker in STEMI. Data from cardiac arrest patients suggest a reduction of copeptin levels through MTH; however, copeptin levels have not been investigated in MTH during STEMI. Methods: We analyzed patients treated with MTH during STEMI in a sub-study of the STATIM trial (Testori, Heart 2019). Patients were randomized to normothermia or MTH with out-of-hospital initiation. Seven copeptin samples were collected from each patient. Primary endpoint was the difference in copeptin levels between the groups. As secondary endpoints, we defined differences in the kinetics between the sampling timepoints and the correlation between copeptin and the infarct size in relation to left ventricular myocardium. Results: We included 99 patients (MTH *n* = 47, control *n* = 52) in our intention to treat analysis. No differences in copeptin values at first medical contact between the MTH and normothermia groups were found. MTH showed no effect on copeptin levels, neither during cooling phase nor through the course. Copeptin peaked at first medical contact and hospital admission in both groups. No differences in kinetics between the timepoints were found. Copeptin showed no correlation with infarct size, neither at first medical contact nor hospital admission. Conclusions: Copeptin levels were not influenced by MTH in STEMI, suggesting the use of this biomarker also during temperature management. Furthermore, copeptin levels were not usable as a surrogate marker for infarct size at any timepoint.

## 1. Introduction

Ischemic heart disease is the leading cause of death worldwide [1]. Primary percutaneous coronary intervention (pPCI) is the gold-standard to reduce myocardial damage in ST-segment elevation myocardial infarction (STEMI) [2]. Cessation of blood flow through plaque rupture and subsequent thrombotic occlusion of a coronary vessel leads to ischemia and, therefore, to myocardial damage. As a result of reopening of the occluded vessel by pPCI, the cytotoxic cascade of reperfusion injury begins to develop. In the effort to reduce the myocardial damage, mild therapeutic hypothermia (MTH) has become one approach to research [3,4,5,6].

Comparable to STEMI, the damage in cardiac arrest occurs in two phases as well. MTH reduces both oxygen demand and neuroexcitatory and inflammatory stress processes after restoration of spontaneous circulation [7] and improves neurological outcome in cardiac arrest patients irrespective of their initial rhythm [8,9]. Therefore, MTH is an established therapy in post-resuscitation treatment [10].

In animal models, MTH is able to reduce myocardial damage [11,12,13]. Several study groups have tried to translate these results to humans. Unfortunately, although rapid cooling is feasible in awake patients [14], most studies have failed to show a reduction in myocardial injury in STEMI [3,4,5,6].

Vasopressin is a peptide hormone released from the pituitary gland together with neurophysin II and copeptin in equimolar amounts. Its release is triggered by osmoreceptors in the hypothalamus, resulting in water reabsorption in the kidney and vasoconstriction. It further stimulates ACTH release, with a subsequent increase in cortisol levels. Consequently, vasopressin improves cardiac output and activates a hormonal stress reaction [15]. As vasopressin is small and highly unstable, the more durable copeptin is used as a surrogate for vasopressin levels in clinical practice [16].

Over the past decade, copeptin has evolved as a biomarker of global circulatory stress in several diseases, including STEMI. It is released very early after symptom onset and is, therefore, used in some strategies for early rule-out together with troponin [17]. In cardiac arrest, it was shown that absolute copeptin levels were lower in patients treated with MTH (33 °C vs. normothermia) irrespective of their neurological outcome [18].

Therefore, we investigated the effect of MTH on copeptin in STEMI.

## 2. Methods

### 2.1. Study Design and Population

The primordial STATIM study was a randomized, controlled, open-label, endpoint blinded trial conducted in accordance with the declaration of Helsinki. Detailed information about the randomization and informed consent process is given in the original publication [6]. In brief, patients between 18 and 75 years with ST-elevation myocardial infarction and symptom onset between 30 min and 6 h were included and randomized either to MTH (34 °C) or normothermia.

Patients with a history of myocardial infarction, acute or chronic heart failure (defined as New York Heart Association or KILLIP Class > I), cardiac arrest, tympanic temperature of less than 35 °C, clinical signs of infection, hepatic failure, recent stroke, hematological dyscrasias, oral anticoagulant treatment or severe pulmonary disease were excluded.

After randomization to the MTH group, patients received buspirone (Busp, Hexal, Holzkirchen, Germany) and meperidine (Alodan, GL Pharma, Lannach, Austria) for anti-shivering.

MTH was initiated in the pre-hospital setting with cold saline (20 mL/kg for inferior STEMI, 10 mL/kg for anterior STEMI). Subsequently, cooling pads (EMCOOLS Flex.Pad, EMCOOLS Emergency Medical Cooling Systems, Pfaffstätten, Austria) were placed on the patient’s trunk and thighs. Before pPCI was performed, an endovascular cooling catheter (Accutrol 14Fr catheter linked with an InnerCool RTx endovascular console; ZOLL Medical, Chelmsford, MA, USA) was placed into the inferior vena cava via the femoral vein, with the tip at the level of the diaphragm. Target temperature was set to 34 °C and maintained for 60 min after reperfusion. After passive rewarming, the cooling catheter was removed at 36 °C body core temperature.

The first copeptin sampling was performed at first medical contact in the out-of-hospital setting prior to any study related interventions. After initiation of cooling, further samples were collected at hospital admission, prior to pPCI and 1 h after pPCI. Additional measurements after rewarming were performed at 12, 24 and 72 h. Plasma samples were centrifuged at the earliest possible date for 15 min and stored at −80 °C until analysis. We used B.R.A.H.M.S. Copeptin proAVP KRYPTOR immunofluorescence assay (B.R.A.H.M.S. GmbH, Thermo Fisher Scientific, Hennigsdorf, Germany) for measurement of copeptin with a sensitivity of 0.41 pmol/L. All assays were performed following the manufacturer’s instructions.

Cardiac MRI was performed 4 ± 2 days after pPCI with a 1.5 T system (Avanto Fit, Siemens Medical Systems, Erlangen, Germany). The detailed protocol is described elsewhere [6].

As no improvement in myocardial salvage attributable to MTH was found in our original trial, we consider our data appropriate to study independent effects of MTH on copeptin levels.

### 2.2. Endpoints

Primary endpoint was the effect of MTH on copeptin levels in STEMI. Kinetics (delta values) between the sample timepoints and the correlation between copeptin at first medical contact or hospital admission and infarct size in relation to left ventricular myocardium (%) served as secondary endpoints. Subgroup analyses for this potential correlation were performed for MTH only and anterior wall infarction.

### 2.3. Statistical Analysis

Categorical variables are presented as absolute count and relative frequency, continuous variables as mean and standard deviation. Copeptin was log transformed to yield an approximately normal distribution. To compare baseline copeptin values between groups, the independent sample t-test was used. We calculated differences from baseline values and present mean and between-individual standard deviations. To compare the change values of copeptin from baseline (delta) between groups, allowing for the panel data structure with repeated measurements, a linear random effects model with patient ID as the cluster identifier was used. Pearson’s correlation coefficient was used to test for correlations. Data management and analyses were performed with MS Excel and Stata 14. A two-sided *p*-value < 0.05 was considered statistically significant.

## 3. Results

During the study period, 120 patients were randomized either to MTH or the control group. After exclusion of 19 (16%) patients due to randomization errors and two (2%) patients due to a lack of copeptin levels, 99 patients remained for intention to treat (ITT) analysis (MTH group *n* = 47, control group *n* = 52).

Baseline characteristics were balanced between intervention and control groups, except for prior statin use (MTH 3/47, control 11/52; *p* = 0.035) and opioid administration (Table 1). The latter was driven by the study design.

### 3.1. Baseline Characteristics

**Figure 1:**
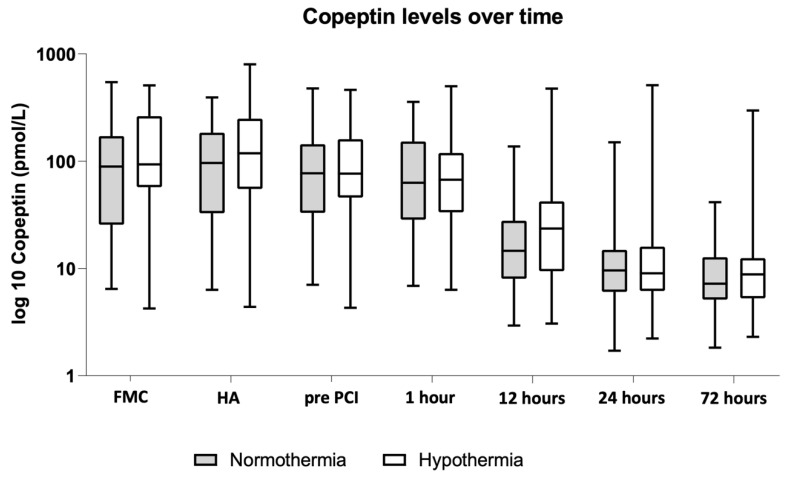
Copeptin levels over time. FMC: First Medical Contact; HA: Hospital Admission; pre PCI: before Percutaneous Coronary Intervention. Data are log 10 transformed. At first medical contact, no differences in copeptin values between the MTH (101.225 pmol/L (57.84–258.13 pmol/L)) and control group (89.11 pmol/L (25.6–170.9 pmol/L), *p* = 0.209) were found.

Target temperature (35 °C prior to reperfusion) was reached in 38/47 patients (81%) in the MTH group. Copeptin values for different sampling timepoints are presented in Table 2 and Figure 1.

### 3.2. Copeptin and Troponin T Levels during the Treatment

In a linear random effects model, MTH showed no statistically significant effect on copeptin levels during the cooling phase (95% CI −0.229–0.570, *p* = 0.403). Also, over all timepoints, no effect was found (95% CI −0.143–0.476, *p* = 0.290).

MTH had no effect on copeptin kinetics, neither during the cooling phase (95% CI −72.204–27.575, *p* = 0.381) nor over the whole study period (95% CI −89.798–18.386, *p* = 0.196). We computed all our analysis for the ITT set as well as for only the adequately cooled patients. No differences were found.

Due to its physiological role, copeptin may be influenced by arterial hypertension or intake of diuretics. Therefore, we computed our random effects model with consideration of those covariates. No effect was found for hypertension (95% CI −0.048–0.600, *p* = 0.095) or prior use of diuretics (95% CI −0.945–1.290, *p* = 0.762).

No correlation was found between copeptin levels and infarct size in relation to left ventricular myocardium on day 4 ± 2 at first medical contact (r = −0.005, *p* = 0.679, data available for 61 patients) or at hospital admission (r = 0.011, *p* = 0.308, data available for 85 patients). We further performed subgroup analyses for at hospital admission for MTH only (r = 0.012, *p* = 0.349) and anterior wall infarction (r = 0.011, *p* = 0.444) parallel to the above-described studies.

## 4. Discussion

In this sub-study of a randomized controlled trial, we could show that copeptin levels were not significantly influenced by MTH in STEMI. Copeptin peaked early in both groups, with its maximum at first medical contact and hospital admission followed by a rapid drop over the next 24 h. Neither differences in the kinetics between sampling timepoints nor absolute numbers were found to be statistically significant. Copeptin at first medical contact or hospital admission was not correlated with infarct size in relation to left ventricular myocardium.

### 4.1. Copeptin as Ubiquitous Biomarker for Circulatory Stress

As explained, copeptin is mainly used as a surrogate for vasopressin, although it is still under debate whether copeptin is by itself hormonally active [15]. Beside its role in the diagnosis of acute coronary syndromes, copeptin is of predictive value in traumatic as well as in non-traumatic settings [19,20]. Krychtiuk et al. further showed that copeptin predicts mortality on admission to intensive care units in general [21]. Accordingly, copeptin has turned out to be a ubiquitous pituitary biomarker of hormonal and circulatory stress. Although MTH as therapy is able to reduce stress-induced cell death, we could not detect an indirect effect on copeptin in our data. One explanation could be that MTH indeed reduces cell stress but the additional burden through the therapy―cooling of awake patients―may outweigh this effect. Although patients were continuously controlled for shivering, it cannot be ruled out that subclinical shivering led to more stress in this group. Conversely, data from Eitel et al. suggest a potential positive impact of opioids on infarct size and reperfusion injury in STEMI patients [22]. Although this effect was only seen in early presenters (≤120 min), central nervous effects of opioids may impact infarct size, and that may have diminished the effect of MTH. There is also evidence that opioids inhibit vasopressin release from the pituitary gland in rats [23]. In vivo, there are conflicting data as to whether the inhibition of vasopressin cells is modulated via κ- or µ-receptors [23,24]. In vitro, the inhibitory effects seem to be primarily mediated via κ-receptors [23]. Both meperidine and morphine exert their effects mainly via the µ-opioid receptor but also have κ-receptor affinity. However, the two substances have different binding affinity to µ-receptors [25]. This may lead to a direct effect of the study-related drug therapy. Even though opioids were given to nearly every patient (98% vs. 94%, *p* = n.s.) in both groups, the respective substances differed. As meperidine prevents shivering more effectively than morphine, it was the drug of choice in our study, similarly to earlier trials [3,4,5]. However, this resulted in a more frequent use in the MTH group. Due to the different receptor affinities, it cannot be excluded that a potential effect was hidden by the differing drugs.

In earlier animal studies, it was shown that vasopressin (and, therefore, copeptin) is released not only from the pituitary gland but also directly from the heart. Hupf et al. induced wall stress in isolated rat hearts with consecutive vasopressin induction and release to the coronary vessels [26]. Based on these data, a potential systemic effect of “cardiac” vasopressin and copeptin has been hypothesized. Thus, the equal levels in our study groups were explained at least partially. However, Boeckel et al. demonstrated that in human patients with STEMI there is no myocardial copeptin release [27].

### 4.2. Kinetics

Copeptin peaked early in our data in both groups. The highest values were measured at first medical contact and hospital admission, which is in accordance with earlier investigations [28]. Copeptin is released immediately after symptom onset before other, conventional biomarkers appear in plasma. Morgenthaler et al. found copeptin in healthy volunteers at a median concentration of 4.2 pmol/L (range 1–13.8 pmol/L). In our study, copeptin dropped almost to its normal range within the first 24 h, as was previously reported [28]. As no statistically significant differences were found between the MTH and control groups in our study, copeptin seems to be an appropriate marker also during the use of temperature management.

### 4.3. Infarct Size

In our study, no correlation between infarct size in relation to left ventricular myocardium and copeptin was found at any sampling timepoint. Previous studies analyzed for this correlation with inconclusive results. Ananth et al. showed a statistically significant correlation between infarct size and copeptin levels in 60 patients [29]. In another relatively small study, also two days after admission, a positive correlation between copeptin and infarct size was found [30]. By contrast, a recently published sub-study of the DANAMI-3 trial with 468 patients found no association between copeptin and area at risk [31].

### 4.4. Strengths and Limitations

To our knowledge, this is the first study analyzing the influence of hypothermia on copeptin values in STEMI. Due to the randomized, controlled design of the original study, baseline characteristics were balanced in both groups. Reflecting the early peak of copeptin, we collected our first samples already in the pre-hospital setting. Current research emphasizes the necessity of early copeptin measurements to improve the diagnostic value, such as in the ongoing AROMI trial [32]. By the exclusion of patients with stroke, chronic heart failure and signs of infection, which are all associated with copeptin release, we minimized the risk of distortion of our investigated concentrations.

Nevertheless, we have some limitations to consider. As this trial included only hemodynamically stable patients, our results cannot be applied to patients in cardiogenic shock with increased circulatory stress and subsequent higher copeptin levels. Another limitation concerns the relatively small number of patients under observation. However, the original study was designed to detect a clinically relevant difference in myocardial salvage, which is why we assume the sample size to be sufficient. Comparable to our study, Broessner et al. had a relatively small sample size (*n* = 134) in their investigation of effects of MTH on copeptin [18]. Unfortunately, neither information about the used analgesics (i.e., opioids) nor about the rate of prescribed statins is available from this trial. Thus, it is not possible to compare possible drug effects. Furthermore, we cannot exclude that MTH was too mild to cause biologically relevant effects.

## 5. Conclusions

Our results show that copeptin levels were not influenced by MTH in STEMI. Copeptin peaked early in both groups, with comparable kinetics for both hypothermia and normothermia, but did not correlate with infarct size. This facilitates the usage and interpretation of this biomarker in further studies concerning MTH in STEMI.

## Figures and Tables

**Table 1:** Baseline characteristics.

	Hypothermia (*n* = 47)	Control (*n* = 52)
Age, years (SD)	58 (±10)	56 (±10)
Female sex, n (%)	10 (21)	9 (17)
Hypertension, n (%)	13 (28)	23 (44)
Diabetes, n (%)	5 (11)	10 (19)
Dyslipidemia, n (%)	10 (21)	16 (31)
Current smoker, n (%)	26 (55)	29 (56)
Family history of CAD, n (%)	12 (26)	12 (23)
Obesity, n (%)	10 (21)	19 (37)
Body mass index (kg/m^2^), mean (SD)	27.0 (±5.5)	29.5 (±4.9)
Previous medication		
Acetylsalicylic acid, n (%)	1 (2)	4 (8)
Beta blocker, n (%)	4 (9)	6 (12)
ACE-I/ARB, n (%)	8 (17)	9 (17)
Statin, n (%) *	3 (6)	11 (21)
Diuretics, n (%)	1 (2)	1 (2)
Initial sinus rhythm, n (%)	44 (94)	48 (92)
ECG diagnosis of anterior wall infarction, n (%)	27 (57)	24 (46)
Emergency therapy		
Acetylsalicylic acid, n (%)	46 (98)	52 (100)
Heparin, n (%)	46 (98)	52 (100)
Prasugrel/Ticagrelor	47 (100)	51 (98)
Nitroglycerin, n (%)	9 (19)	12 (23)
Beta blocker, n (%)	0 (0)	3 (6)
Opioids, n (%)	46 (98)	49 (94)
Morphine, n (%) ^†^	11 (23)	44 (85)
Meperidin, n (%) ^†^	42 (89)	5 (10)
Infarct-related artery		
Left anterior descending artery, n (%)	28 (60)	23 (44)
Circumflex artery, n (%)	3 (6)	4 (8)
Right coronary artery, n (%)	16 (34)	25 (48)
Multivessel disease, n (%)	22 (47)	30 (58)
Initial TIMI grade flow 0/1, n (%)	34 (72)	42 (81)

* *p* = 0.035, † *p* < 0.001.

**Table 2:** Copeptin values as pmol/L [IQR] and Troponin T values as ng/L [IQR].

Copeptin	Hypothermia (*n* = 47)	Control (*n* = 52)	*p*-Value
First medical contact	101.3 (57.8–258.1)	89.1 (25.6–170.9)	0.216
Hospital admission	118.5 (55.1–248.6)	96.2 (32.7–184.2)	0.148
Prior to pPCI	76.5 (45.9–160.2)	77.1 (33.1–143.6)	0.353
1 h after pPCI	67.2 (33.4–119.2)	62.7 (28.5–152.0)	0.636
12 h after pPCI	23.5 (9.4–42.1)	14.6 (8.0–27.8)	0.157
24 h after pPCI	9.0 (6.2–15.9)	9.6 (6.1–14.9)	0.710
72 h after pPCI	8.8 (5.3–12.4)	7.2 (5.1–12.7)	0.742
**Troponin T**			
Hospital admission	43 (18–103)	24.5 (17–58.25)	0.182
Troponin peak level	4480 (1750–6746)	3610 (2112.5–6800)	0.969

## Data Availability

For further data and material please contact the corresponding author.
